# Innovative Community-Based Approaches Doubled Tuberculosis Case Notification and Improve Treatment Outcome in Southern Ethiopia

**DOI:** 10.1371/journal.pone.0063174

**Published:** 2013-05-27

**Authors:** Mohammed A. Yassin, Daniel G. Datiko, Olivia Tulloch, Paulos Markos, Melkamsew Aschalew, Estifanos B. Shargie, Mesay H. Dangisso, Ryuichi Komatsu, Suvanand Sahu, Lucie Blok, Luis E. Cuevas, Sally Theobald

**Affiliations:** 1 Global Fund to Fight AIDS, Tuberculosis and Malaria, Geneva, Switzerland; 2 Liverpool School of Tropical Medicine, Liverpool, United Kingdom; 3 TB REACH Project, Sidama zone, Hawassa, Ethiopia; 4 Southern Region Health Bureau, Hawassa, Ethiopia; 5 TB REACH, Stop TB Partnership, Geneva, Switzerland; 6 Royal Tropical Institute (KIT), Amsterdam, The Netherlands; McGill University, Canada

## Abstract

**Background:**

TB Control Programmes rely on passive case-finding to detect cases. TB notification remains low in Ethiopia despite major expansion of health services. Poor rural communities face many barriers to service access.

**Methods and Findings:**

A community-based intervention package was implemented in Sidama zone, Ethiopia. The package included advocacy, training, engaging stakeholders and communities and active case-finding by female Health Extension Workers (HEWs) at village level. HEWs conducted house-to-house visits, identified individuals with a cough for two or more weeks, with or without other symptoms, collected sputum, prepared smears and supervised treatment. Supervisors transported smears for microscopy, started treatment, screened contacts and initiated Isoniazid preventive therapy (IPT) for children. Outcomes were compared with the pre-implementation period and a control zone. Qualitative research was conducted to understand community and provider perceptions and experiences.

HEWs screened 49,857 symptomatic individuals (60% women) from October 2010 to December 2011. 2,262 (4·5%) had smear-positive TB (53% women). Case notification increased from 64 to 127/100,000 population/year resulting in 5,090 PTB+ and 7,071 cases of all forms of TB. Of 8,005 contacts visited, 1,949 were symptomatic, 1,290 symptomatic were tested and 69 diagnosed with TB. 1,080 children received IPT. Treatment success for smear-positive TB increased from 77% to 93% and treatment default decreased from 11% to 3%. Service users and providers found the intervention package highly acceptable.

**Conclusions:**

Community-based interventions made TB diagnostic and treatment services more accessible to the poor, women, elderly and children, doubling the notification rate and improving treatment outcome. This approach could improve TB diagnosis and treatment in other high burden settings.

## Introduction

Tuberculosis (TB) is one of the major causes of morbidity and mortality in Ethiopia. Despite the introduction of the Directly Observed Therapy Short course (DOTS) strategy in the 1990 s and its expansion to most health facilities by 2010, the number of notified cases in the country is still low compared to the estimated number of cases and has been stable for the last 10 years. TB Case notification rates were 45 and 47 per 100,000 population and treatment success rate of smear-positive TB cases were 80% and 83% in 2000 and 2010, respectively [1]. Innovative interventions to increase case detection and to improve treatment outcome are therefore needed. The Ethiopian National TB Control Program (NTP) relies on passive case finding among symptomatic individuals visiting health facilities. However, as over 80% of the population live in rural areas and most TB diagnostic and treatment centres are located in urban areas, many patients need to travel to access these relatively distant health facilities. Patients often make several visits for diagnosis and to receive treatment [2], [3], [4], [5], which become onerous, particularly for poor and vulnerable individuals such as women, children and the elderly [6], [7], [8]. In addition, fear of stigma and poor knowledge about the disease and the availability of treatment contribute to individuals either delaying or failing to access health services [5], [9].

Ethiopia introduced a Health Service Extension Program (HSEP) in 2003 to improve access to essential health services through community-based services delivered by Health Extension Workers (HEWs) [10]. The program provides preventive and curative activities as 16 health packages targeting households, and particularly women and children, at *kebele* level, the smallest administrative unit with a population of about 5000. HEWs are salaried females trained for one year by the HSEP and come from and live within the communities they serve. Their work is implemented by conducting house-to-house visits and staffing the *kebele’s* health post. HEWs are supported by community health promoters (CHPs), who are lay volunteers selected by the community. The HSEP activities on TB are limited to the creation of awareness of TB, referral of symptomatic cases and advice on treatment adherence [11].

Although HEWs could be further engaged to improve TB case detection and treatment outcome [12], there are no studies to demonstrate whether this approach is feasible in a programmatic setting or acceptable to the community. We therefore describe here the experience of implementing an intervention package engaging HEWs to improve TB case detection and treatment outcome in Ethiopia.

## Methods

**Figure 1:**
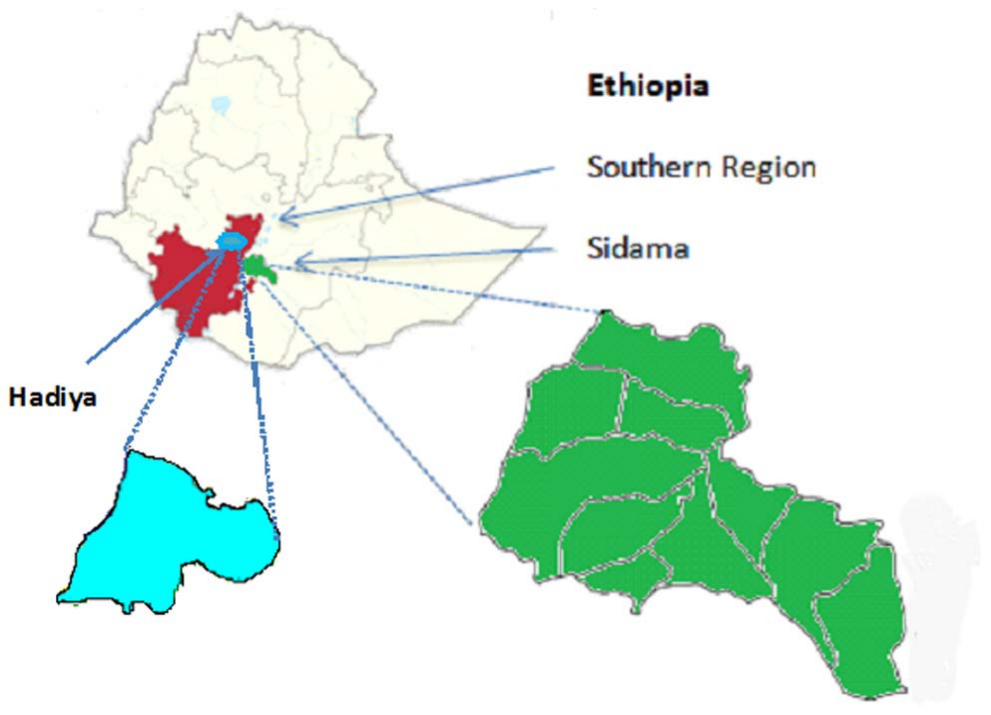
Map of the implementation zone (Sidama) and the 19 districts and the control zone (Hadiya).

This was an implementation study evaluating the introduction of an innovative intervention package in Sidama zone, in the Southern Region of Ethiopia, as shown in Figure 1. The intervention aimed to improve TB case detection and treatment outcome. Sidama zone has 19 administrative districts, a population of over 3 million and is served by 2 public hospitals, 109 health centres and 7 clinics.

**Figure 2:**
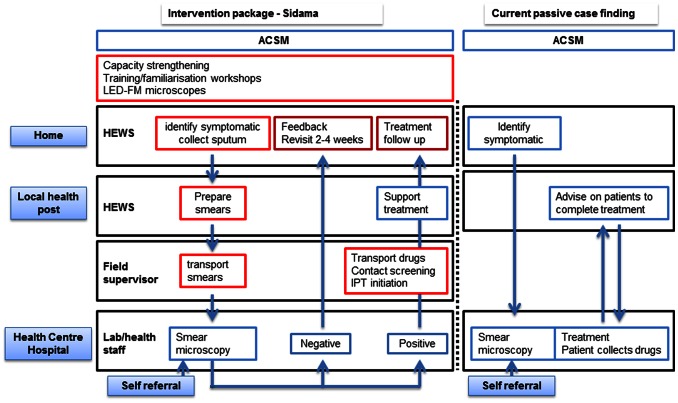
Schematic representation of the intervention package and the current passive case finding approach.

Table 1 and Figure 2 outline the key components of the implementation package:

**Table 1:** Components of the implementation package.

**Familiarization and awareness** **creation workshops**	At zone, district and community level. Attended by political, community and religious leaders, teachers, stakeholders, partners, health workers, HEWs and ex-TB patients.
**HEW training**	Covered TB and the new community-based approach including; identification of TB symptomatic individuals, sputum collection, quality assessment, preparing and fixing smears at community level, universal precautions for infection control and slide storage, recording and reporting, treatment support and follow-up and drug side effects.
**Staff and laboratory technician** **training and microscope** **distribution**	Refresher training for staff involved in TB control activities and district supervisors. Fluorescent microscopes (light-emitting diodes – LED-FM) were distributed to laboratories (one per district and one for the Reference laboratory). Laboratory technicians received training on FM staining, smear grading and external quality assessment (EQA) procedures.
**ACSM activities**	Key messages about TB and availability of services within the communities were conveyed during community meetings, campaigns and through local radio.
**HEWs Identified people having** **cough for two or more weeks** **(Active case finding)**	House-to-house visits, informing symptomatic individuals to produce and submit good quality sputum samples, preparing and fixing smears in the health posts. Two sputum specimens were collected on-the spot and the next morning. Patients’ information recorded on the slides and logbooks and HEWs used mobile phones to contact supervisors to arrange transportation of smeared slides.
**Processing slides**	Supervisors collected slides from HEWs and transported them to health centers. Laboratory technicians stained smears using Ziehl-Neelsen and/or Auramine and examined under light or Fluorescent microscopes, grade smears based international and kept slides for EQA and recorded patients’ details in the laboratory register. Laboratories performing LED-FM received training and on-site visits from senior laboratory technologists and were certified to do fluorescent microscopy.
**Treatment initiation and** **screening household contacts**	Supervisors collected results from laboratory technicians, registered patients and initiated anti-TB treatment for PTB+ cases in their residences. Supervisors also screened household contacts of PTB+ cases (all household contacts living in the same shelter/house with the index PTB+ cases) and initiated Isoniazid preventive therapy (IPT) for asymptomatic children aged less than 5 years. Symptomatic children were referred for further examination (e.g. X-ray).
**Treatment monitoring**	HEWs supported and monitored treatment, reported drug side-effects and treatment outcome, followed smear-negative cases, collected sputum samples again or referred patients to health centers/hospitals for further investigation.
**Routine Recording and** **Reporting system**	Supervisors supported the routine recording and reporting systems, updated registers and harmonized results with TB focal persons.
**Feedback**	Quarterly reports submitted to the NTP/MOH and TB REACH for feedback; feedback from EQA shared with laboratory technicians.
**M&E**	Regular review meetings were conducted with NTP staff, district supervisors and HEWs to discuss achievements, challenges and follow-up actions. Activities and performance were monitored by the field team and the NTP and an independent M&E expert contracted by TB REACH evaluated the performance and feedback used to improve quality and performance. Routine quarterly surveillance data were collected from the control zone and any change/new interventions related to TB control activities were monitored and documented.

1. A capacity strengthening component with tailored training workshops for Health Bureau, HEWs and laboratory staff; familiarization and awareness creation workshops for political, community and religious leaders, teachers and other stakeholders; the provision of light-emitting-diodes-fluorescent microscopes (LED-FM) for laboratories and the appointment of one project supervisor per district to support and supervise field activities.
2. An advocacy, communication and social mobilization (ACSM) component delivering messages about TB and the availability of the services during community meetings, campaigns and the local radios.
3. An active case finding component, in which HEWs conducted house-to-house visits to identify individuals with chronic cough, informed symptomatic individuals how to produce sputum and preparation of smears of two sputum specimens for smear microscopy.
4. A communication and transport component, with the provision of mobile phone airtime fees to all HEWs and supervisors for communication, motorbikes for supervisors to transport smeared-slides and to return test results and treatment.
5. A treatment component, with the provision and monitoring of home-based treatment, screening of household contacts to identify and investigate symptomatic individuals, Isoniazid preventive therapy (IPT) for asymptomatic children <5 years old and follow up of smear negative cases.

The package was implemented in close collaboration with the Regional Health Bureau and the NTP. The effect of the package was assessed by comparing the number of symptomatic individuals screened, the number of cases notified and the treatment outcome at baseline and during the implementation period (October 2010 to December 2011). Hadiya zone, which did not receive the intervention package, was used as a control zone to compare the outcomes. Hadiya zone, which did not receive the intervention package, was used as a control zone to compare the outcomes. Hadiya zone has similar characteristics to the intervention zone; it has a population of 1,355,153, 46 TB diagnostic and treatment centres and in 2010, 924 smear-positive TB cases were notified with case notification rate of 68 per 100,000 population and 82% of TB patients were successfully treated.

Changes in program implementation and potential confounders; including expansion of TB diagnostic and treatment units in the intervention zone, intensified/active case finding or opening new TB diagnostic and treatment units in the control Zone and disruption of laboratory supplies or TB medicines in the intervention or control Zone were documented and monitored. The number of health facilities providing TB diagnostic and treatment services remained the same during the study period. Retrospective surveillance data collected from the TB registers of health facilities providing TB treatment and the quarterly surveillance reports from Sidama zone for the period between June 2009 and September 2010 were used as baseline.

In addition, a qualitative study was conducted in seven districts to understand the experiences and views of the provider and community about the package. These districts were purposively selected to capture diverse distances from the project office and rural and urban populations. In-depth interviews were undertaken with community members screened for TB by HEWs (n = 21), HEWs and CHPs (n = 20), supervisors (n = 5) and laboratory technicians (n = 14). Focus group discussions (FDGs) were conducted with HEWs, supervisors and laboratory technicians (n = 5). FGDs participants were selected purposively to capture maximum diversity regarding sex, age, socio-economic status and geography and years of experience. Interviews were conducted in the vernacular, recorded, transcribed and translated into English. The quality of translations was monitored by the project’s senior staff. Quality was assured through joint assessment of the sampling approach and on-going review of transcripts to explore areas for further probing.

### Statistical Analysis

The data were entered and analysed using SPSS package and the impact of the package was assessed by comparing the number of cases detected and treatment outcome before and after implementation and between the intervention and control zones using summary statistics with 95% confidence intervals (95% CI); Chi-square to test differences in proportions. P values <0·05 were considered statistically significant.

Qualitative data was analysed thematically using a framework approach, which is an open accountable approach allowing the inclusion of multiple analyses. [13] Transcripts were coded in Ethiopia and the UK using Nvivo (QSR NVivo version 9·2). The interviews’ coding tree and interpretations were discussed and triangulation was undertaken by method and participant group. Commonly used concepts were cited.

Support for the implementation of the project was obtained from the Ministry of Health of Ethiopia and the Southern Region Health Bureau. Ethical approval was obtained from the Research Ethics Committee of the Liverpool School of Tropical Medicine, UK (protocol number 10.69). Verbal consent was obtained from all individuals who participated in the qualitative studies, as indicated in the ethical approval. Written consent was not always feasible due to the low level of literacy in rural communities. Following standard procedure and approved by the ethics committee, illiterate participants were read the information sheet and their consent was observed and signed by a witness, audio records were retained. Programmatic data were collected from the TB control formats and registers and used for secondary data analysis. In all cases, confidentiality of participants was retained and only limited number of staff had access to individual's identifiers.

## Results

### Training

The package was implemented in 524 *Kebeles* of the 19 districts. Implementation started through a public launch and training in October 2010. Familiarization and awareness creation workshops were attended by 1,200 participants. One HEW per *kebele* (n = 524), 19 HEWs supervisors and 300 staff of public health facilities were trained.

### Identification of Symptomatic Individuals and Diagnosis

**Figure 3:**
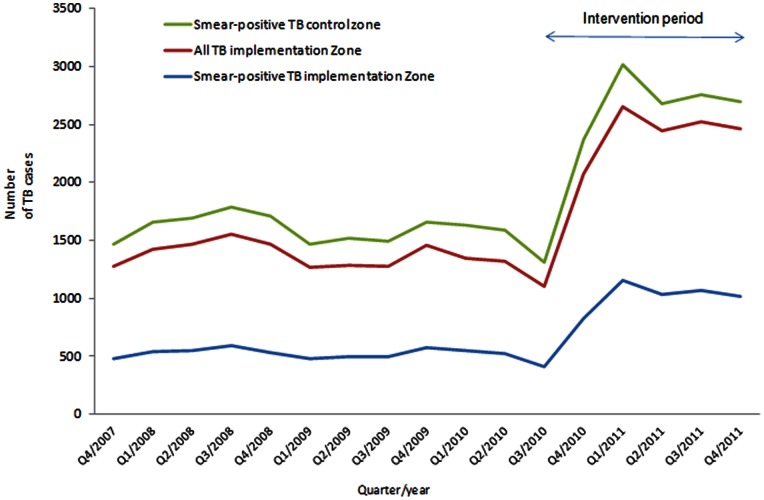
Trends in number of TB cases detected before (quarter 4, 2007 to quarter 3, 2010) and during the implementation period (from quarter 4, 2010 to quarter 4, 2011) in the intervention and control zones.

Between October 2010 and December 2011, HEWs identified 49,857 (29,314 [60%] women) individuals with cough for two or more weeks, with or without other symptoms. Of these, 2,262 (4·5%) (1,199 [53%] women) were smear-positive (PTB+), as shown in tables 2 and 3. The male to female ratio among PTB+ cases changed from 1.3∶1 before the intervention to almost 1∶1. Children <15 years old represented 12% (5,727) of symptomatic individuals and 9% (178) of PTB+ cases. Similarly, people >55 years old represented 10% (5,103) of symptomatic individuals and 8% (169) of PTB+ cases, as shown in table 4. In addition, 2,828 PTB+, 922 smear-negative TB (PTB-) and 993 extra-pulmonary TB (EPTB) cases were diagnosed by the public health facilities. The proportion of women among PTB+ cases was lower in the public health facilities than in the community (44% and 53%; P<0·001). In total 5,090 PTB+ and 7,071 cases with all forms of TB were diagnosed during the intervention period. In comparison 2,534 PTB+ and 3,968 cases of all forms of TB had been diagnosed during the 15-month baseline period (June 2009 to September 2010), corresponding to an increase of 101% and 78%, respectively. The number of PTB+ cases in the control zone was 949 prior to the project and 1,133 during the project period (+16% increase, 95% CI = 14^.^5–18^.^5). The case notification rate (95% CI) in the intervention zone therefore increased from 64 (62·5–65·8) to 127 (123·8–131·2) PTB+ cases per 100,000 population per year. Similarly, notification rates for all forms of TB increased from 102 (99·1–105·8) to 177 (172·6–181·0) cases per 100,000 population, as shown in Table 2 and Figure 3.

**Table 2:** Comparison of TB screening and diagnosis in implementation zone prior and during the implementation periods.

Activity	Baseline	Project period	Additionality	Remarks
	(June 2009–Sep 10)	(Oct 2010–Dec 2011)		
TB symptomatic individuals screened at community level	0	49,857	49,857	60% women
PTB+ cases detected at community level	0	2,262	2,262	54% women
TB symptomatic individuals screened in the zone	12,800	58,909	46,109	58% women
All PTB+ cases detected	2,534	5,090	2,556	
PTB+ case notification rate (per 100,000) per year (95%CI)	64 (62·5–65·8)	127 (123·8–131·2)	63	
All forms of TB diagnosed and initiated treatment	3,968	7,071	3,102	46% women
Contact traced (screened)	0	8,005 (1,290)	8,005	2,906 index case
TB cases among contacts	0	69	69	62 PTB+, 28 females
Contact children age <5 years old offered IPT	0	1,080	1,080	
IPT completion rate	0	92% (643/698)	643	Jul-Dec 2011 cohort
Treatment success rate (PTB+)	77%	93%	16%	
Defaulter rate (PTB+)	11%	3%	8%	

**Table 3:** Type of TB screening used and proportion of PTB+ cases detected and registered for treatment using different case finding approaches.

Main outcomes	Symptomaticindividuals tested	PTB+ detected	PTB+ casesinitiated treatment
Case finding by HEWs (active case finding)	49,857	1262 (4·5%)	1262 (100%)
Case finding by health facilities (passive case finding)	9000	2828 (31%)*	2828 (100%)
Case finding among contacts of PTB+ cases (by supervisors and HEWs)	1290	62 (4·8%)	62 (100%)

PTB+ = Smear-positive pulmonary Tuberculosis, HEW = Health Extension Workers;

* Smear-positive TB cases referred from other centres included.

**Table 4:** Age and sex distribution of new PTB+ cases diagnosed between October 2010 and December 2011 in Sidama zone.

Reporting period	Sex	0–4	5–14	15–24	25–34	35–44	45–54	55–64	≥65	Total
Quarter 1	M	1	25	151	115	51	38	27	13	421
	F	0	45	123	111	43	22	7	7	358
Quarter 2	M	3	37	175	149	75	75	16	15	545
	F	3	51	128	184	69	64	24	15	538
Quarter 3	M	0	43	149	127	64	57	32	14	486
	F	2	51	138	169	75	44	17	8	504
Quarter 4	M	0	44	150	141	76	40	40	20	511
	F	2	52	121	150	93	60	18	8	504
Quarter 5	M	3	41	165	138	74	63	33	28	545
	F	2	38	109	138	65	41	18	5	416
All	M	7	190	790	670	340	273	148	90	2508
	F	9	237	619	752	345	231	84	43	2320

External quality assurance (EQA) of smear microscopy was conducted for all smear microscopy centres. Thirty-two (1·5%) of 2073 slides collected had discordant grades between the peripheral and the reference laboratories.

### Identification of Household Contacts

Eight thousand and five household contacts of PTB+ cases were visited by the HEWs and supervisors. Of these, 1,949 (13%) had cough for two or more weeks, with or without other symptoms and 1,290 (66%) provided sputum samples. Sixty-two (4·8%) of 1,290 were PTB+ and seven PTB- or EPTB based on clinical and X-ray findings. Twenty-eight of the 69 cases among contacts were females, 16 (25%) of PTB+ and all PTB- and EPTB cases were children <15 years old. A total of 2,477 household contacts were children, of which 1,080 (44%) were under <5 years old and asymptomatic and were offered IPT. Of 698 children provided with IPT between July and December 2011, 643 (92%) had completed the preventive treatment and 54 (8%) interrupted treatment and one child developed active TB.

### Treatment Registration and Outcome

All 5,090 (100%) patients with TB diagnosed in the intervention zone initiated treatment during the implementation period. Of 1,850 new PTB+ cases registered in quarter 1 and 2, 1716 (93%, [95% CI 91·8–94·2] were successfully treated; 85% cured, 8% completed treatment), 2% (41) died, 3% (52) defaulted, 0·1% (two) failed treatment and 2% (39) were transferred out/not evaluated. In comparison, of 1,921 PTB+ cases registered during the 15 months prior to the implementation period, 1476 (77%, 95% CI 75·0–78·8) had been successfully treated (42% cured, 35% completed treatment), 3% (46) had died, 11% (219) defaulted, 0·3% (6) failed treatment and 9% (174) were transferred out/not evaluated (p<0·05 for treatment success, defaulter and cases not evaluated), as shown in table 5. The treatment success rates of smear-negative, EPTB and retreatment cases during the implementation period were 91%, 91% and 92%, respectively, compared to 75%, 76% and 67% respectively prior to the implementation period (p<0·05 for all), as shown in table 5.

**Table 5:** Treatment outcome of cohorts registered one year prior to the project period and during the first two implementation quarters.

Category	Cured	Completed	Treatmentsuccess	Died	Failure	Defaulter	Transferredout/not evaluated	Registered
**Baseline (August 2009–Sep 2010)**								
New PTB+	810 (42)	666 (35)	1476 (77)	46 (2)	6 (0·3)	219 (11)	174 (9)	1,921
PNEG	0 (0)	352 (75)	352 (75)	13 (3)	0 (0)	66 (14)	40 (9)	471
EPTB	0 (0)	411 (76)	411 (76)	21 (4)	2 (0·4)	67 (12)	42 (8)	543
Re-treatment	56 (32)	63 (36)	119 (67)	13 (7)	0 (0)	28 (16)	17 (10)	177
**Intervention period (October 2010 to March 2011)** *								
New PTB+	1,578 (85)	138 (8)	1,716 (93)	41 (2)	2 (0·1)	52 (3)	39 (2)	1,850
PNEG	0	357 (91)	357 (91)	10 (3)	0	17 (4)	10 (3)	394
EPTB	0	347 (91)	347 (91)	9 (2)	0	13 (3)	11 (3)	380
Re-treatment	81 (68)	29 (24)	110 (92)	1 (1)	0	3 (3)	5 (4)	119

* patients registered after March 2011 were still on treatment.

### Community and Provider Perspectives

The implementation of the package depended on committed HEWs, supervisors and collaboration with the TB control program. The qualitative studies established there was a high level of acceptability from service users and providers, with enthusiasm and passion for the package.

*Symptomatic individuals/TB patients* had learnt about TB from HEWs’ and CHPs’ mobilization activities and found “community/home-based services” highly acceptable and convenient. Some participants indicated they would have been unable to get a diagnosis without the intervention due to direct and opportunity costs and would have “waited at home for death”. Respondents often referred to multiple barriers to diagnosis faced pre-intervention. For example distance was particularly challenging for women, the poor, elderly and the very sick. Community-based treatment reduced difficulties associated with adherence, although lack of food remained an important issue for some patients.

*Providers* described commitment or “devotion” to improving the health of their communities who lacked education on health matters, yet accepted guidance through the ACSM activities, and highlighted the package improved access and awareness, particularly for the very poor and women. HEWs felt job satisfaction collecting and preparing smears, the preventive and curative aspects of their work and felt guided and supported by supervisors. Being a HEW involved in “TB work” warranted “respect” from the community.

*Laboratory technicians* felt the intervention as a whole was very beneficial, the number of people diagnosed had increased and accessibility to “forgotten people” had improved. They had experienced a substantial increase in the number of slides to be examined but acknowledged the financial incentives received for the extra work. Technicians also felt they had benefitted from new equipment but complained about the quality of the smears prepared by HEWs in the early stages of implementation and the level of training received. Some were proactive in visiting communities and assisting HEWs in preparing slides with support from the supervisor and reported subsequent quality improvement.

## Discussion

This community-based TB intervention package brought diagnostic and treatment services closer to poor rural communities; women, children and vulnerable groups particularly benefited. The outcome measures and analysis of providers’ and service users’ experiences confirm that making TB services available at community level through engaging HEWs improves access and service utilization. The implementation of the package doubled the notification rate of PTB+ cases compared to the pre-project period and to the control zone. This sharp increase is likely to be attributable to the intervention, as there were no other factors identified that could explain the increase.

Many studies report that men are more frequently diagnosed as having TB and have higher smear positivity rates than women [14], [15], [16], [17]. Possible explanations include women experiencing barriers to service access [18], longer clinical delays in diagnosis [19] or producing sputum of poor quality [20]. In the intervention zone the male to female ratio among PTB+ cases was close to 1∶1 and significantly more women were diagnosed with TB at community level than in the health facilities. The interventions have reduced barriers to services with poor women who had previously faced difficulties travelling to health centres particularly benefitting. The proportions of children and elderly among symptomatic and PTB+ cases also increased during the implementation period, and these are also vulnerable groups better reached by an intervention package that is embedded in the community.

As diagnosis of PTB- and EPTB require health facility visits, their increase during the implementation period was marginal compared to PTB+; as most of the PTB+ cases were detected at community-level.

Despite a substantially increased cohort of patients, all patients who were diagnosed started treatment. Treatment outcomes also improved significantly with the implementation of the package. Over 90% of all TB cases registered were successfully treated and defaulter rate decreased by almost two-thirds. Several studies have reported that the main reasons for defaulting from TB treatment are distance between patients’ residence and the DOTS centres, socio-economic and cultural factors and lack of support and awareness [21], [22]. The qualitative findings showed that the extensive ACSM activities promoted openness about TB, with patients being positive about the opportunity for both diagnosis and treatment at community level.

Contact investigation is a key intervention to improve case detection; reduce the risk of transmission and increase the likelihood of diagnosing other cases among close contacts. [23], [24] Although contact tracing and IPT for children are recommended by the NTP [11], they were not implemented in the routine TB programme in the region prior to the package implementation. IPT was initiated during the second half of the project implementation and was quickly scaled-up with over 1000 children within 6 months. The service was decentralized to the community level and attained high uptake and adherence with 92% completing treatment. A previous study in a neighbouring zone (Hawassa) had shown that IPT uptake in children was poor with only 12% of those initiating IPT completing the recommended 6-month course [25].

The implementation package also contributed to capacity building and improved the routine TB recording and reporting systems through regular supportive supervision. It has ensured data quality (completeness, consistency and timeliness) and introduced and scaled up EQA for smear microscopy. The quality of smears prepared by HEWs improved through on-site training and regular feedback loops. The proportion of discordant slides between the readings in the peripheral laboratories and the reference centre remained low and was within the acceptable range.

In a context of human resource constraints there is a renewed recognition that Community Health Workers (CHWs) are an integral component of the health workforce needed to achieve the Millennium Development Goals in many low and middle income countries [26]. Although many countries are currently implementing this concept, there is a high level of variation in the characteristics of the cadres enlisted and the resources and packages included in its implementation. Female HEW cadres in Ethiopia have a unique opportunity to support improved access to health services, including active case finding for TB and treatment support by operating at the community level. The intervention project staff worked in partnership with the Ethiopian HSEP, emphasised training, close supervision and support for HEW. HEWs reported job satisfaction and positive feedback from the community; were motivated to support their communities and welcomed training and supervision from the district field supervisors.

Assessing the perspectives of communities and stakeholders using qualitative research supports the ongoing delivery and sustainability of interventions by capturing the perspectives and motivations of different stakeholders. Detailed understanding of the motivations of HEWs in this context is important for future adaptations of this promising approach in other contexts and further research is needed to demonstrate the cost-benefit and long term performance of the intervention.

Despite the limitations of the study, in which the package was not implemented in an experimental study design, we have shown that the decentralization of TB diagnostic and treatment services and the involvement of HEWs in TB control is feasible, leads to substantial gains in case detection and care, and is key to building more equitable and improved TB control efforts in Ethiopia. The components of the package were synergistic and its success was supported by working within the existing health system and linking the strategy within the on-going TB control programme and the government-initiated community-based health extension program. This approach should also foster sustainability and scale up through the full integration of the package into the HSEP and the NTP. The approach has the potential for national scale-up and for adaption in other resource-poor high TB burden settings.
